# When Alcoholism Affects Memory Functions: MRI of the Brain

**Published:** 1995

**Authors:** Terry L. Jernigan, Arne L. Ostergaard

**Affiliations:** Terry L. Jernigan, Ph.D., is a staff psychologist in the Department of Veterans Affairs Medical Center, San Diego, and associate professor in the departments of psychiatry and radiology, University of California San Diego, School of Medicine, San Diego, California. Arne L. Ostergaard, Ph.D., is an assistant adjunct professor in the Department of Psychiatry, University of California San Diego, School of Medicine, San Diego, California

**Keywords:** AOD dependence, memory, magnetic resonance imaging, brain, AODR (alcohol and other drug related) amnestic syndrome, AOD impairment, scientific model

## Abstract

The development of modern imaging techniques makes it possible to examine directly the relationship between brain abnormalities and memory impairment. Alcoholic amnesics may perform normally on certain tests (priming tasks) that require implicit (unconscious) memory, even though they may not be able consciously to recall the memory. Researchers have therefore postulated the existence of multiple memory mechanisms. Magnetic resonance imaging (MRI) observations suggest that independent memory mechanisms are not necessary to explain the dissociation between explicit and implicit memory. Alcoholic amnesics appear to suffer from damage to structures in two areas of the brain, affecting two separate processing components that are both required in most priming tasks: a stimulus processing component and a memory processing component.

The development of modern imaging techniques makes it possible to examine the brains of subjects who are at different stages of alcoholism and whose medical and mental status can be ascertained at the time of imaging. Thus it is possible to examine directly the relationship between brain abnormalities and cognitive impairment and to screen patients carefully for other medical conditions that may contribute to such impairment.

This article summarizes research on memory deficits in alcoholics, emphasizing evidence obtained using brain imaging methods. Recent neurological theories about human memory are outlined, and relevant evidence is presented from brain imaging studies of alcoholics in which damage to specific brain structures is linked to particular aspects of memory performance.

## Brain Imaging

The past two decades have witnessed the development of increasingly refined techniques for viewing the living brain. Computed tomography (CT) uses rotating x-ray beams to measure differences in tissue density across thin sections of brain tissue. CT provides excellent images of fluid-filled spaces in the brain, enabling researchers to determine the overall degree of brain tissue shrinkage associated with alcoholism or other disorders (Fox et al. 1976). Although CT is more sensitive to gradations of tissue density than a conventional skull X ray, it does not distinguish clearly between white and gray matter. Therefore it cannot delineate individual anatomical structures within the brain.

**Figure 1:**
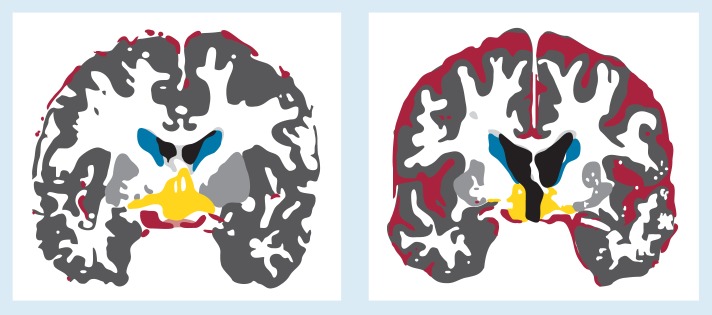
Magnetic resonance images. A brain section from a normal subject (left) is compared with the same section from an alcoholic subject (right). Certain features have been color-coded to facilitate comparison. The amount of cerebrospinal fluid (bright red) in the grooves on the brain surface (sulci) is increased in both amnesic and nonamnesic alcoholic subjects compared with normal subjects. The cerebral ventricles, or fluid-filled cavities within the brain (black), are larger in alcoholic subjects—more so in amnesic than in nonamnesic alcoholic subjects. In addition, the caudate nucleus (blue), part of the corpus striatum, and a section of the diencephalon (yellow) are smaller in alcoholic amnesics.

Increased size of fluid-filled brain structures, such as the cerebral ventricles^1^ and cortical sulci, is evidence of brain degeneration. However, this finding alone reveals little about the specific brain structures that are affected by this degeneration. The size of individual brain structures can be determined using magnetic resonance imaging (MRI) (figure 1). In MRI, the patient is placed in a chamber and exposed to radio waves in the presence of a powerful magnetic field. Different types of tissue produce different energy signals in response to this procedure (Pfefferbaum et al. 1990). MRI scanners translate the signals into three-dimensional images that depict specific anatomical structures in fine detail. MRI is therefore a very sensitive tool for detecting and localizing small abnormalities of brain tissue. Measurements obtained from MRI images of patients can then be compared to those from normal controls.

## Alcohol and Memory

One of the most common forms of severe amnesia associated with alcohol abuse is Wernicke-Korsakoff syndrome. The acute phase of the syndrome (Wernicke’s encephalopathy) is characterized by mental confusion, eye movement abnormalities, and poor muscular coordination (for review, see Butters and Cermak 1980). When patients are treated with the B vitamin thiamine, their neurologic symptoms generally improve. However, most patients who recover from Wernicke’s encephalopathy are left with a severe residual memory impairment called Korsakoff syndrome. The hallmark of this syndrome is a striking loss of the ability to learn new things. Although patients appear to comprehend what is going on around them, they may forget events moments after they have occurred. These symptoms are associated with anatomical damage to the limbic system, a brain region comprising several structures on the mesial (inner) surface of the temporal lobe, and to structures in the midline of the diencephalon (Victor et al. 1989).

### Mental Processes That Support Memory

Learning a new telephone number, recalling the right word to use in conversation, recognizing a face, and finding one’s way in a familiar neighborhood are generally considered functions of memory. However, these tasks all are assisted by information processing functions that are not memory processes per se. Among these information processing functions are the ability to focus and maintain attention and perception, and the ability to recognize and identify stimuli such as objects, figures, and words. Memory processes include learning and retrieving data.

This distinction is important because long-term alcoholism is associated not only with learning and retrieval deficits, but also with moderate declines in the ability to process information to be remembered (for more information, see the article by Evert and Oscar-Berman, pp. 89–96). Thus, poor performance on memory tests may reflect deficits in memory, attention, or comprehension. Only when a patient has difficulty learning and retrieving information that is disproportionate to his or her ability to attend to, recognize, and identify the information does the psychologist infer a memory deficit per se.

### Priming

Despite disabling memory problems, alcoholic amnesics learn some things well. For example, many of these patients can acquire new motor skills (e.g., tracking a moving object) as quickly as nonamnesics. This suggests that motor skill learning may employ neural mechanisms different from learning of facts and events. Some evidence suggests that alcoholic amnesics also can learn certain cognitive skills (e.g., a mirror reading task) normally (Cohen and Squire 1980).

Perhaps most surprisingly, memory for words and pictures may be normal in amnesics, although at an unconscious level. This phenomenon of unconscious remembering can be demonstrated by priming experiments. Priming in this context refers to the effect of previous exposure on the processing of a stimulus. In a typical priming test, a subject must read aloud different words that appear sequentially on a screen. The experimenter measures the time between each word’s appearance and the subject’s response. Subjects generally respond more quickly to words previously presented (primed words) compared with words not previously presented (novel words). The priming effect appears to occur whether the subject consciously remembers having seen the words before or not. Priming studies show that the processing of stimuli by alcoholic amnesics is significantly improved by recent prior exposure and that this effect is similar in magnitude to that obtained in subjects with normal memory.

## Theory of Multiple Memory Systems

Evidence exists (Butters et al. 1990; Heindel et al. 1991) that dementing illnesses like Alzheimer’s disease, which destroy limbic and neocortical structures, often result in impaired priming but normal skill learning. On the other hand, illnesses like Huntington’s disease, which destroy basal ganglia (e.g., striatal structures) are associated with impaired skill learning but normal priming.

All these findings have been integrated into a comprehensive neurological theory of human memory that posits the existence of multiple, independent memory systems in the brain. This theory attributes explicit memory to the mesial temporal lobe and diencephalic brain structures, skill learning to the striatum, and priming to the neocortex (see Squire 1986 and Jernigan and Cermak 1994 for reviews).

This model, combined with the pattern of memory performance observed in alcoholic amnesics, makes certain predictions about the sites of brain abnormality in these patients. Given the severe explicit memory deficits they experience, damage is expected in the system that includes the mesial temporal lobe structures and related structures in the midline diencephalon. However, because previous research has emphasized how learning skills are spared and the occurrence of priming, the striatum and neocortex are expected to be unaffected. In the following discussion, the direct anatomical evidence will be summarized and compared to these predictions.

## Anatomical Correlates of Alcoholic Amnesia

In a study of 28 male nonamnesic alcoholics, MRI images of the brain were subjected to detailed anatomical analysis (Jernigan et al. 1991*a*). Results were compared with those from a group of 36 matched nonalcoholic men. The amount of cerebrospinal fluid in the cerebral ventricles and in the cortical sulci was significantly increased in the alcoholic subjects, indicating loss of brain tissue. Individual brain structures were examined to determine whether the volume loss was generalized or limited to specific regions. The diencephalon, the caudate nucleus (a component of the striatum), and parts of the cerebral cortex and the limbic system (including the mesial temporal lobe) were significantly smaller in alcoholic subjects. Subjects exhibited mild deficits on attentional and perceptual-motor tasks. However, they did not have significant memory deficits of the type observed in alcoholic amnesics (e.g., recalling ideas from a story after a 30-minute delay).

A second study, using the same MRI methods, compared a group of eight alcoholic amnesics to matched nonamnesic alcoholics (Jernigan et al. 1991*b*). The alcoholic amnesics demonstrated a very similar pattern of brain volume losses to the nonamnesic alcoholics. However, the amnesics had significantly greater losses in specific midline diencephalic structures, the mesial temporal region, and the forward portion of the cortex above the eyes. The more severe memory problems afflicting these patients may therefore be attributable to damage in some of the structures within these regions.

These anatomical studies of subjects with alcoholic amnesia are in some ways consistent with predictions based on the multiple systems theory of human memory. Subjects exhibited damage in both the diencephalic and mesial temporal lobe components of the brain system thought to control explicit memory. Thus it is not surprising that these patients have severe deficits on tests of recognition and recall. However, if the multiple systems theory is correct, it *is* surprising that impairments in skill learning and priming rarely have been observed in alcoholic amnesics, because these patients do seem to have damage in striatal and neocortical structures as well. Therefore, the observation of striatal and cortical damage in alcoholic amnesics is inconsistent with the multiple systems model used to explain their memory performances. On the other hand, such damage is consistent with the evidence, described earlier, of alcohol-related deficits in stimulus processing and perceptual-motor function because these functions also have been associated (like priming and skill learning) with neocortical and striatal brain systems.

The results of a recent study of priming and explicit memory function in memory-impaired subjects (Jernigan and Ostergaard 1993) may help to reconcile these inconsistencies. A group of 30 subjects with explicit memory performance ranging from normal to severely impaired was examined with tests of word priming and word recognition. Priming was measured as an improvement in the ability to identify words presented very briefly. Word identification performance for previously studied words was compared to performance for unstudied words. The difference in performance was taken as the measure of priming, or implicit memory. Explicit memory was measured by mixing studied with unstudied words and asking the subjects to state whether or not each word had been presented earlier in the test. Of this group, some of the subjects were alcoholic amnesics, some had amnesias attributable to other causes, some had dementing illnesses, and some were healthy controls. Concurrent to the behavioral testing, MRI was performed and the anatomical methods described previously were applied.

As expected, within this group of subjects, the greater the damage to the mesial temporal lobe structures the greater the impairment of recognition memory. However, the magnitude of the priming effect was not correlated with the degree of neocortical damage. Instead, striatal and mesial temporal lobe damage both appeared to affect priming, but in opposite ways. That is, striatal damage was associated with larger priming effects, whereas mesial temporal damage was associated with smaller priming effects. Subjects with both striatal and mesial temporal damage exhibited intermediate priming effects.

To a large extent, the observed relationship between striatal damage and increased priming effects may be an artifact—a reflection of the experimental design rather than the subjects’ inherent priming abilities. Some of the test items may be so easy to process that priming does not improve performance scores, even if prior exposure to the item did produce a strong memory. Thus, measurements of priming alone may not adequately represent memory, especially when the subject already is performing well on the task without being primed.

Within this context, the results of the anatomical study of memory impairment make more sense. Because subjects without striatal damage processed new (non-primed) words efficiently, their priming scores were misleadingly low. Because subjects with striatal damage processed new words inefficiently, they had more room for improvement; thus, their priming scores more closely reflected the strength of the memory that resulted from the prior exposure. If the patients with striatal damage had normal memory, their priming scores were higher than the priming scores of subjects without striatal damage. If they had poor memory, their priming scores approached, or were even lower than, those of subjects without striatal damage.

If the above explanation is correct, the results of studies of priming in patients with alcohol-related memory impairment should be reconsidered; the distinction between the kind of memory measured as priming and the kind measured in explicit tasks may be spurious. Alcoholic amnesics may appear to perform normally on priming tasks largely because the priming scores of normal control subjects, used for comparison, are misleadingly low.

If the above explanation is accepted, the observation of normal priming in the context of impaired explicit memory no longer requires the existence of an independent memory mechanism for priming (see Ostergaard and Jernigan 1993, for review). It is suggested that priming, like recognition memory, is affected by damage to the mesial temporal lobe/diencephalic system. However, on many priming tasks, measured priming also will be related to proficiency at the baseline task, which may be reduced by striatal damage and perhaps by damage to other structures as well. Dissociation between priming and explicit memory is therefore likely to occur in alcoholic amnesics because they suffer from damage to both striatal and limbic structures in the brain. Damage in these two sites appears to affect two separate processing components, both of which are required in most priming tasks: One is a stimulus processing component and the other is a memory processing component.

## Future Research Directions

Further research will be necessary to determine whether the multiple systems theory or the multiple processes theory described above is a better explanation of the nature of the alcoholic amnesia syndrome. A variety of implicit and explicit memory tests should be administered to subjects with alcohol-related memory deficits. Results of such studies should reveal whether the apparent dissociations between amnesics’ performance on these two types of memory tasks are generally associated with poorer baseline performance. Other factors that influence the extent to which memory strength is reflected in priming scores also may play a role.

Some of the questions raised in this article may be resolved by using functional brain imaging techniques to compare patterns of brain activity between subjects performing explicit memory tasks and subjects performing implicit tasks, such as priming. Brain activity patterns also should be compared between nonalcoholic and alcoholic subjects with and without amnesia.

## Footnotes

1 For definitions of this and other technical terms, see glossary, pp. 136–137.
